# Electrostatic Potential Analysis in Polyelectrolyte Brush-Grafted Microchannels Filled with Polyelectrolyte Dispersion

**DOI:** 10.3390/mi12121475

**Published:** 2021-11-29

**Authors:** Byoungjin Chun, Myung-Suk Chun

**Affiliations:** 1 Complex Fluids Laboratory, Advanced Materials Research Division, Korea Institute of Science and Technology (KIST), Seoul 02792, Korea; byoungjin.chun@gmail.com; 2 Department of Chemical and Biological Engineering, Korea University, Seoul 02841, Korea; 3 Biomedical Engineering Department, KIST School, Korea University of Science and Technology, Seoul 02792, Korea

**Keywords:** microchannel, microfluidics, polyelectrolyte solution, polyelectrolyte brush, Poisson–Nernst–Planck equations, electrostatic potential, charge density, ion transport, continuum modeling

## Abstract

In this study, the model framework that includes almost all relevant parameters of interest has been developed to quantify the electrostatic potential and charge density occurring in microchannels grafted with polyelectrolyte brushes and simultaneously filled with polyelectrolyte dispersion. The brush layer is described by the Alexander-de Gennes model incorporated with the monomer distribution function accompanying the quadratic decay. Each ion concentration due to mobile charges in the bulk and fixed charges in the brush layer can be determined by multi-species ion balance. We solved 2-dimensional Poisson–Nernst–Planck equations adopted for simulating electric field with ion transport in the soft channel, by considering anionic polyelectrolyte of polyacrylic acid (PAA). Remarkable results were obtained regarding the brush height, ionization, electrostatic potential, and charge density profiles with conditions of brush, dispersion, and solution pH. The Donnan potential in the brush channel shows several times higher than the surface potential in the bare channel, whereas it becomes lower with increasing PAA concentration. Our framework is fruitful to provide comparative information regarding electrostatic interaction properties, serving as an important bridge between modeling and experiments, and is possible to couple with governing equations for flow field.

## 1. Introduction

Polymer brushes are widely used to control the physical and chemical properties of solid surfaces, according to changes of molecular structure and chemical composition [1,2,3]. The reconstruction of bulk polymers results in long response times (several minutes to tens of hours), since various polymer constituents either migrate to the surface from the bulk or rearrange locally. On the contrary, the grafted polymer brush and self-assembled multilayered films have the advantage of a tunable and fast response to external stimuli, ranging possibly from a few seconds to hours [4,5,6,7]. The rapid stimuli-responsive surfaces allow us to use them in a diverse range of applications (e.g., drug delivery, biosensors, diagnostics, and actuators) that are based on the mechanism of thermo-responsive [8,9], pH-responsive [10,11,12], redox-responsive [13,14], and so on.

The polyelectrolyte (PE) should be distinguished from the neutral polymer due to its ability to dissociate charges in solvents, resulting in a charged polymer chain (macro-ion) and mobile counter-ions. The PE brush plays an important role in flow fields at narrow spaces, for example, the critical effects of flow-induced deformation of the PE or transport at PE-grafted interfaces in nanochannels where the brush height is comparable to the channel width [15,16,17,18]. For microfluidic channels whose width is much larger than the brush height, changes of flow field are generally observed near the channel wall rather than in the bulk region. Since the surface modifications alter either the electric double layer (EDL) thickness of channel wall or the adsorption energy of suspended particulates, the PE brush-grafted microchannels can be more useful in a variety of chemical and biological applications. In particular, the biological complex fluids are mostly dispersed in aqueous solution, in which suspended particulates coated with polyelectrolytes should be characterized by stabilization and electrostatic surface charge with variations of solution pH. Exchanging surface charges with the surrounding ions in a bulk medium affects the behavior of surface potential (cf., experimentally, zeta potential) as well as ionic conductance [19,20,21].

Among the theoretical descriptions for PE brush properties, the Alexander-de Gennes (AdG) scaling model is applied in the present study, which provides the fundamental platform with conceptual simplicity [22,23,24]. The starting point of the AdG model is the total free energy of a single grafted PE molecules as a sum of the elastic energy originating from chain stretching (which favors a compact coil), excluded volume (which tends to swell), and electrostatic contribution. Subsequently, the brush height is obtained based on scaling law according to the variation of the grafting density. As the grafting density increases, the electrostatic effects become smaller than the other two contributions [25]. When the grafting density is above a critical level such that two adjacent grafted chains start interacting sterically with each other, the electrostatic effects have no bearing on the brush height, indicating that the AdG model for neutral polymer can be reasonably adopted here.

Previous studies on PE brush-grafted nanochannel employed the Poisson–Boltzmann (PB) equation to estimate the electrostatic potential, assuming that the ion concentration within the brush layer follows the Boltzmann distribution [15,16,17,18]. This PB equation has been widely employed in the literature concerning microfluidics in microchannels [26,27,28]. However, the ion concentration within the brush layer depends on the monomer density profile, and the ion concentrations predicted by the Boltzmann distribution can approach infinity with increasing electrostatic potential or surface charge. These implies limited applicability of the Boltzmann distribution in the case of brush-grafted microchannel. Hence, we quantify the electric field with ion transport by the Poisson–Nernst–Planck (PNP) equations, as the general model without requiring (Boltzmann) distribution function.

In this study, the model framework has been developed to analyze the electrostatic interaction behavior in rectangular microchannels with PE brushed wall, by including almost all relevant parameters, such as channel dimension, PE brush conditions, and electrolytic fluid properties. Charge distribution near the channel wall consists of mobile charge in a medium solution and fixed charge in the brush layer. For characterizing the PE brush layer, the AdG model is incorporated with the monomer density profile of brush whereby the general form of the unique parabolic distribution function [24,29] is employed instead of the non-unique cubic distribution function [17]. The prototype channel is considered as polydimethylsiloxane (PDMS) surface grafted with anionic polyacrylic acid (PAA) brush. Both electrostatic potential and charge density with the swelling (i.e., the ratio of the radius of gyration of the perturbed chain to that of the θ-state) of PAA are numerically computed from the PNP continuum model according to variations of solution pH. These data provide useful information to experimental scientists because direct measurements are not possible. As the electrolytic fluids filled inside the channel, we consider the KCl solution as well as the dilute PAA solution dispersed in the KCl solution. In the rheological aspect, PAA dispersion is referred to as a generalized Newtonian fluid exhibiting both Newtonian and shear-thinning behaviors.

## 2. Model Formulations

**Figure 1:**
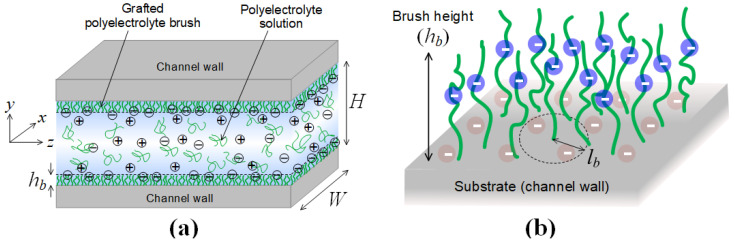
(**a**) Rectangular microchannel with a width *W* and a height *H*, where the inner wall of the channel is covered with grafted PE brush layer; (**b**) PE brushes grafted onto the negatively charged surface with a brush height *h_b_* and an average distance between grafting points *l_b_*.

We consider a rectangular cross-sectional channel consisting of top, bottom, left- and right-sided wall illustrated in Figure 1a, where the inside of the channel surface is covered with anionic PE brush as Figure 1b. Cartesian coordinates are applied for spanwise (*x*), longitudinal (*y*), and streamwise (*z*) distances, respectively. The channel length is much larger than its width and height, so that the variations of electrostatic potential and charge density are neglected along the direction of channel length.

### 2.1. Polyelectrolyte Brush Model with Monomer Density Profile

For describing the PE brush, the Alexander-de Gennes model is used [22,23]. The brush height and monomer density distribution of the brush are varied according to the electrolyte concentration in the bulk solution. As mentioned above, we properly assume a neutral polymer brush with densely grafting condition. The free energy of the polymer brush *F*_brush_ grafted at one end consists of the spring energy of the polymer chain and the excluded volume, as follows:

(1) $$F_{\mathrm{brush}}=\frac{3h_{b}^{2}}{2}\left(\frac{k_{B}T}{N_{K}b_{K}^{2}}\right)+\frac{k_{B}T}{2}\left(\frac{N_{K}^{2}\omega }{h_{b}l_{b}^{2}}\right)\;.$$

Here, *k_B_T*/*N_K_b_K_*^2^ corresponds to the entropic spring constant of the polymer chain with *k_B_T* being the Boltzmann thermal energy (cf. 4.16 × 10^−21^ J at room temperature), *N_K_* being the number of Kuhn segments, and *b_K_* being the length of Kuhn segment (i.e., twice of the persistence length). In Equation (1), *ω* is the excluded volume parameter defined as *b_K_*^3^(1 − 2*χ*) with *χ* being the Flory–Huggins parameter [16,17,25]. With the average distance between grafting points *l_b_*, the brush height at equilibrium *h_b_* can be obtained by minimizing the free energy of polymer chain (i.e., Equation (1)), given by:

(2) $$h_{b}=N_{K}b_{K}{\left[\frac{\left(1-2\chi \right)b_{K}^{2}}{6l_{b}^{2}}\right]}^{1/3}.$$

The monomer distribution has the highest monomer number density at the wall and tends to continuously decrease to the end of PE brush, rather than the uniform form. The monomer distribution is represented as the quadratic decay from the wall [24], expressed in the form:

(3) $$\Phi =\left(N_{K}b_{K}^{3}\sigma /h_{b}\right)\varphi \left(x,y\right)\;,$$

where *N_K_b_K_*^3^*σ*/*h_b_* represents the scaled factor for monomer density. The grafting density *σ* is identified in a unit volume of the brush, given by:

(4) $$\sigma =4/\pi l_{b}^{2}.$$

For cross-section of the rectangular channel, the normalized monomer distribution function *φ*(*x*,*y*) is specified by *φ_x_* along the spanwise (*x*) direction and *φ_y_* along the longitudinal (*y*) direction, and it can be derived as:

(5) $$\begin{array}{ll} \varphi_{x}=\frac{3}{2h_{b}^{2}}\left[h_{b}^{2}-{\left(\left|x\right|-\frac{W}{2}\right)}^{2}\right] & for\;0<\left|x\right|<W/2\;,\\ \varphi_{y}=\frac{3}{2h_{b}^{2}}\left[h_{b}^{2}-{\left(\left|y\right|-\frac{H}{2}\right)}^{2}\right] & for\;0<\left|y\right|<H/2\;. \end{array}$$

The sum of *φ_x_* and *φ_y_* represents *φ*(*x*,*y*) at each corner. Note that the monomer distribution function satisfies the normalization condition by integrating each function over the brush height (or brush layer thickness). Detailed descriptions of the monomer density profile are available in the literature [3,16,24].

### 2.2. Multi-Species Ion Balance in the Bulk Solution

For the surface of the microchannel grafted with PE brushes, the morphology of PE brushes varies with the properties of bulk solution, *inter alia*, a condition of solution pH. Let us consider that background KCl electrolyte, HCl and NaOH for pH adjustment, and a well-known anionic PE of PAA are dissolved in the bulk solution. All of dissociated ions can be listed as:

(6) $$\begin{array}{l} H_{2}O⇌H^{+}+{\mathrm{OH}}^{-}\;,\\ \mathrm{KCl}⇌K^{+}+{\mathrm{Cl}}^{-}\;,\\ \mathrm{HCl}⇌H^{+}+{\mathrm{Cl}}^{-}\;,\\ \mathrm{NaOH}⇌{\mathrm{Na}}^{+}+{\mathrm{OH}}^{-}\;,\\ \mathrm{PAA}⇌n_{s}\left(H^{+}+AA^{-}\right)\;, \end{array}$$

where AA^−^ denotes the acrylate anion (=RCOO^−^), *n_s_* is the number of acrylic acid monomer units in a PAA chain dispersed in the bulk solution. The multi-species ion balance determines molar concentration of ion species *i*, *C_i_* [mM], at a given pH, as follows:

(i) Concentrations of cations: H^+^, K^+^, Na^+^

(7) $$\begin{array}{l} C_{H^{+}}=\;10^{-\mathrm{pH}\;+\;3},\\ C_{K^{+}}=\;\;C_{\mathrm{KCl}},\\ C_{{\mathrm{Na}}^{+}}=n_{s}fC_{\mathrm{PAA}}+10^{-\left(14-\mathrm{pH}\right)\;+\;3}-10^{-\mathrm{pH}\;+\;3}\;\;\left(\mathrm{pH}>7\right)\;. \end{array}$$

(ii) Concentrations of anions: OH^−^, Cl^−^, AA^−^

(8) $$\begin{array}{l} C_{{\mathrm{OH}}^{-}}=\;10^{-(14\;-\;\mathrm{pH})\;+\;3}\;,\\ C_{{\mathrm{Cl}}^{-}}=(\begin{array}{ll} C_{\mathrm{KCl}}{+10}^{-\mathrm{pH}\;+\;3}-10^{-(14\;-\;\mathrm{pH})\;+\;3} & \left(\mathrm{pH}\leq 7\right)\\ C_{\mathrm{KCl}} & \left(\mathrm{pH}>7\right)\;, \end{array}\\ C_{{\mathrm{AA}}^{-}}=\;n_{s}fC_{\mathrm{PAA}}\;. \end{array}$$

Here, the degree of ionization *f* depends on pH of the bulk solution, which can be measured by dissociation experiments in terms of the fractional number of charged monomers in a PAA chain.

**Figure 2:**
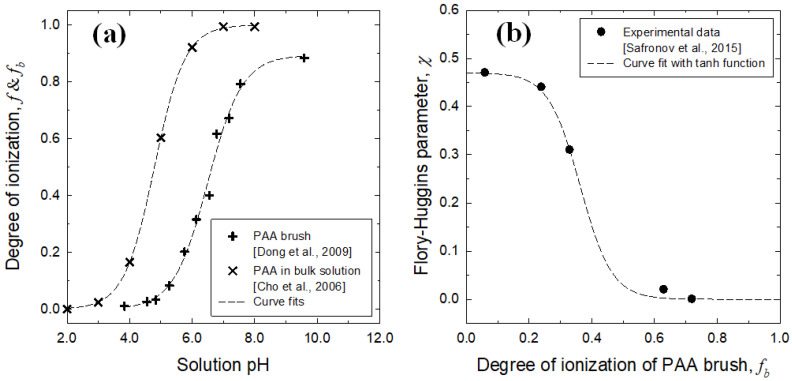
(**a**) The degree of ionization versus solution pH for PAA chain dispersed in the bulk [28] and grafted PAA brush [30], where dashed curves are best fits with sigmoid growth function; (**b**) The Flory–Huggins parameter versus degree of ionization of PAA brush, where symbols indicate the literature data [31] and the dashed curve denotes a best fit to the data with hyperbolic tangent function.

In Figure 2a, the profile from the experiment [30] appears as the S-shaped growth curve by the following fitting function:

(9) pH=pKa−0.49log[(1−f)/f] .

In note, the pKa (=−log Ka) value of PAA in the bulk solution is obtained as 4.79, which is not much different from the literature data of 4.58 [31]. One can see a clear difference between the two curves of *f* by reflecting that the ionization degree of densely grafted PAA brush obviously differs from that of PAA in the bulk solution. For the brush data [32], the fitting curve can be numerically obtained as:

(10) $$\mathrm{pH}={\mathrm{pKa}}_{b}-0.55\log\left[\left(0.89-f_{b}\right)/f_{b}\right]\;,$$

where *f_b_* is the degree of ionization of PAA brush. In this case, the pKa_b_ value is obtained as 6.5, which is much larger than the pKa value. Figure 2a allows us to understand that the PAA chains in the bulk solution undergo high swelling compared to the swelling of PAA brush. We also find that the ionization of PAA brush is incomplete even at higher pH.

The change of brush layer thickness according to the variations of solution pH can be determined by the relationship between the degree of ionization of brush *f*_b_ and Flory–Huggins parameter *χ*. Figure 2b presents the best curve fit to the experimental data found in the literature for different *f*_b_ values [33]. The fitting function is obtained in the form of hyperbolic tangent as *χ* = *a*_1_[1 − tanh(*a*_2_ *f*_b_ − *a*_3_)], where each coefficient is obtained as *a*_1_ = 0.237, *a*_2_ = 9.881, and *a*_3_ = 3.587.

### 2.3. The Poisson–Nernst–Planck Model

When the charged surface is in contact with an electrolyte, the electrostatic charge would influence the distribution of nearby ions so that an electric field is established for locally averaged electrostatic potential *ψ*. For the electrostatic potential distribution in a dielectric material, the volume charge density *ρ_e_* can be represented by the Poisson equation, viz. ∇·(∇*ψ*) ≡ ∇^2^*ψ* = −*ρ_e_*/*ε_o_ε_r_*, where *ε_o_* is the dielectric permittivity of vacuum (=8.85 × 10^−12^ C/V m) and a piecewise constant *ε_r_* is the relative dielectric permittivity of aqueous medium (cf. 78.4 at room temperature). Solvated ions are treated as point charges in the standard mean-field assumption. The mobile charge density in the liquid-filled bare channel can be assumed to follow the Boltzmann distribution, and accordingly the Poisson equation converts to the PB equation quantifying the electrostatic potential distribution and long-range interaction [26].

In the case of PE brush-grafted channel, the Poisson equation is considered as follows:

(11) $$\nabla^{2}\psi =-\rho_{e}^{\mathrm{net}}/\varepsilon_{o}\varepsilon_{r}=-(\rho_{e}+\rho_{\mathrm{fix}})/\varepsilon_{o}\varepsilon_{r}.$$

In Equation (11), the net charge density *ρ_e_*^net^ consists of the mobile charge density *ρ_e_* in a medium solution and the fixed charge density *ρ*_fix_ in the brush layer resulting from ionization of PAA. They are given as, respectively:

(12) $$\rho_{e}=N_{A}e\sum_{i}\Lambda_{i}C_{i},$$

(13) $$\rho_{fix}=N_{A}e\Lambda_{{\mathrm{AA}}^{-}}C_{{\mathrm{AA}}^{-}}.$$

In Equation (12), *N_A_* is the Avogadro number (=6.022 × 10^23^/mol), *e* is the elementary charge (=1.602 × 10^−19^ C), and *Λ_i_* is the valence of ion species *i*. In Equation (13), the molar concentration of acrylate anion in the brush is taken as *C*_AA_^−^ = *n_b_ f_b_ C*_PAA_brush_ with the number of acrylic acid monomer units *n_b_* in a single chain of PAA brush. Here, the brush concentration can be expressed as:

(14) $$C_{\mathrm{PAA}\_\mathrm{brush}}=\sigma \varphi \left(x,y\right)/N_{A}h_{b}\;.$$

The ion transport in the microchannel is described by convection, diffusion, and migration contributions on the basis of the so-called Nernst-Planck (NP) equation [34], as given:

(15) $$J_{i}=\;-\upsilon_{i}C_{i}+D_{i}\nabla C_{i}+\Lambda_{i}eK_{i}C_{i}\nabla \psi \;.$$

Here, **J***_i_* represents the ionic flux vector with ***v***_i_ being the ionic velocity, *D_i_* being the ionic diffusivity, and the mobility of ion species *i* (*K_i_*) equals to *D_i_*/*k_B_T* by the Einstein relation. Coupling the electrostatic potential by the Poisson equation with the NP equation invokes the aforementioned PNP equations. The continuity for ion species *i* under steady state can be written as:

(16) $$\nabla \cdot J_{i}=0\;.$$

One should keep in mind that the Poisson equation and NP equation are accurate in the transport of dilute electrolytic fluids of pointlike ions, due to the inter-ion correlation and effect of ion size occurring in the higher concentration. When the bulk solution is bounded by channel wall in *x* and *y* direction without flow by external force (i.e., ***v***_i_ = 0), Equation (15) can be simplified by noting Equation (16) as follows:

(17a) $$D_{i}\left[\frac{\partial^{2}C_{i}}{\partial x^{2}}+\frac{\Lambda_{i}e}{k_{B}T}\frac{\partial }{\partial x}\left(C_{i}\frac{\partial \psi }{\partial x}\right)\right]=0,$$

(17b) $$D_{i}\left[\frac{\partial^{2}C_{i}}{\partial y^{2}}+\frac{\Lambda_{i}e}{k_{B}T}\frac{\partial }{\partial y}\left(C_{i}\frac{\partial \psi }{\partial y}\right)\right]=0.$$

Equations (17a) and (17b) are to be solved subject to the boundary conditions: (i) −**n**·**J**_*i*_ = 0 at the channel wall (cf., **n** is the unit normal vector directed into the channel surface) as a requirement of no flux through the wall, and (ii) *ψ* = 0 at the bulk region as symmetric about channel centerline.

**Figure 3:**
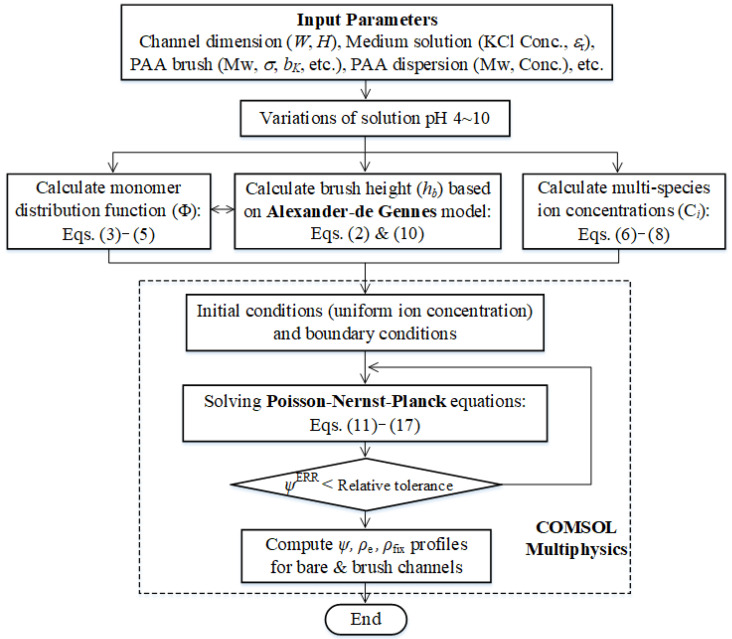
The framework of the numerical algorithm employed in this study.

Overall computation procedures and algorithm are presented in Figure 3. 2-dimensional PNP continuum model needs to guarantee the convergent solution by applying iterations with tolerance of 10^−3^. Our model framework was solved with implementation of the COMSOL Multiphysics (Ver. 5.2a, COMSOL Inc, Burlington, MA, USA) with a finite element method [35].

## 3. Results and Discussion

As the prototype channel for computations, rectangular-slit geometry is considered with the width *W* of 8 *μ*m and the height *H* of 2 *μ*m having channel aspect ratio *W*/*H* of 4, and this channel dimension is sufficiently larger than the EDL thickness and the brush height. The EDL thickness (or Debye length) is defined as *κ*^−1^ = [*ε_o_ε_r_k_B_T*/2*N_A_e*^2^*I*]^1/2^ for electrolyte solution, where the ionic strength *I* is ∑*_i_ C_i_Λ_i_*^2^/2. The background fluid is the monovalent electrolyte of 0.01 mM KCl solution, and PE dispersion is modeled as the PAA dispersed in this KCl solution with concentrations of 0.2, 0.4, and 0.8 wt%. The pH of KCl solution and PAA solution is adjusted by HCl and NaOH. The EDL thickness *κ*^−1^ is estimated as 96.5 nm for background fluid of 0.01 mM KCl at pH 7, but *κ*^−1^ decreases distinctly with increasing either PAA concentration or solution pH.

To provide an insight for realistic predictions, rigorous values of material parameters are required. In the case of bare channel without covering the brush, the value of surface potential of PDMS for various pH can be obtained from the literature [27,36,37]. The molecular weight (Mw) of PAA for PE dispersion is 45 kDa, whereas the PAA brush is considered as the Mw of 30 kDa and the Kuhn segment length *b_K_* of 0.64 nm [38,39,40]. Although electrostatic potential and charge density profiles are computed for the entire channel cross-section, we present only the exemplar results near the bottom wall.

### 3.1. Brush Layer and Bulk Solution Analysis

**Figure 4:**
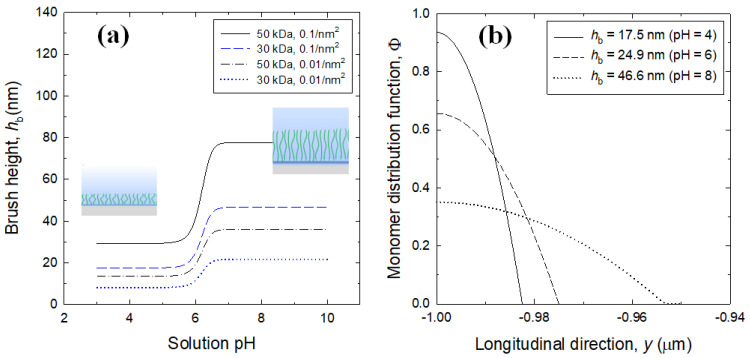
(**a**) The estimated PAA brush heights with variations of solution pH for different molecular weights and grafting densities; (**b**) The profiles of monomer distribution function within PAA brush layer near the bottom wall (−*H*/2 ≤ *y* ≤ −*H*/2 + *h_b_*) for Mw = 30 kDa and *σ* = 0.1/nm^2^.

In Figure 4a, the brush height increases sharply between pH 6 and 6.5 but remains constant elsewhere. At the same pH, the brush height increases with higher Mw or higher grafting density. This behavior is more pronounced with swollen brushes above pH 6.5, because of the greater excluded volume effect on the brush segments. Figure 4b shows the parabolic profiles of monomer density function for different solution pH, and each profile demonstrates the quadratic decay that occurs from the maximum value at channel wall to the end of the brush upon a solid-liquid interface. The grafting density of 0.1/nm^2^ allows us to recognize that two adjacent grafted chains can interact enough with each other. PAA brush chains are almost neutral at low pH according to very weak ionization, but swell with increasing pH due to an increase of the electrostatic repulsion between anionic segments.

**Figure 5:**
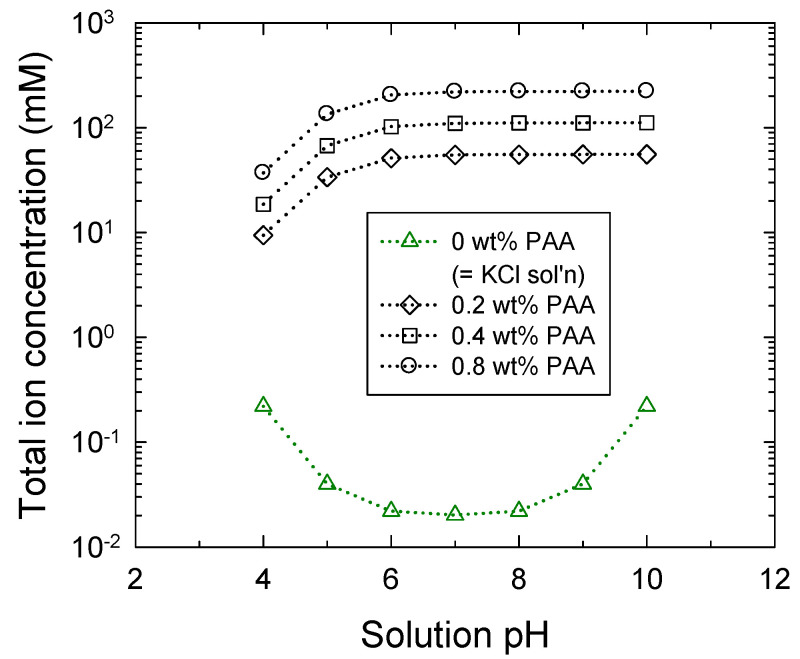
The total ion concentration in the bulk solution with variations of pH and PAA concentrations of 0, 0.2, 0.4, and 0.8 wt%, where 0 wt% PAA means 0.01 mM KCl solution.

In Figure 5, total ion concentration in the bulk solution increases with increasing PAA concentrations from 0 to 0.8 wt%. Its change in KCl solution (e.g., 0 wt% PAA) is symmetric with respect to pH 7, where the minimum is 0.02 mM as the sum of K^+^ and Cl^−^ concentrations. It needs to be emphasized that PAA solutions obtain total ion concentrations several tens to 10^4^ times higher than that of the KCl solution, which is caused by ionization of the acrylic acid. Their total ion concentrations increase with increasing solution pH.

### 3.2. Electrostatic Potential and Charge Density Profiles

**Figure 6:**
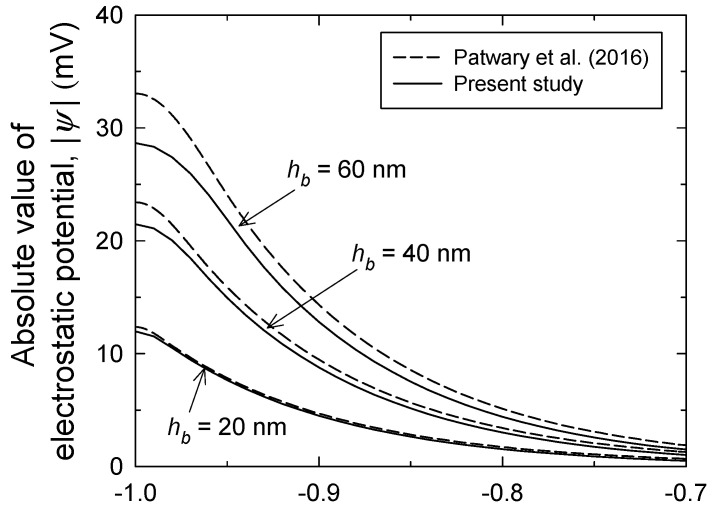
Electrostatic potential profiles near the PE brush-grafted channel wall with various brush heights for 0.01 mM KCl solution of pH 5 and pKa = 4. Solid and dashed curves indicate our results and literature results [17], respectively. Here, the literature results were replotted ones, by solving the provided model equations.

As provided in Figure 6 for different brush heights, we compared our results computed from the PNP model with the results in the literature obtained by the PB equation [17]. Both results have a qualitatively identical trend, in which the electrostatic potential profiles exhibit a slow decline near the wall, but decrease exponentially outside the brush layer, implying the contribution of Donnan potential within the brush layer. There is no discrepancy between two results when *h_b_* is 20 nm, but the previous result based on Boltzmann distribution appears to predict a higher potential profile with increasing *h_b_*.

**Figure 7:**
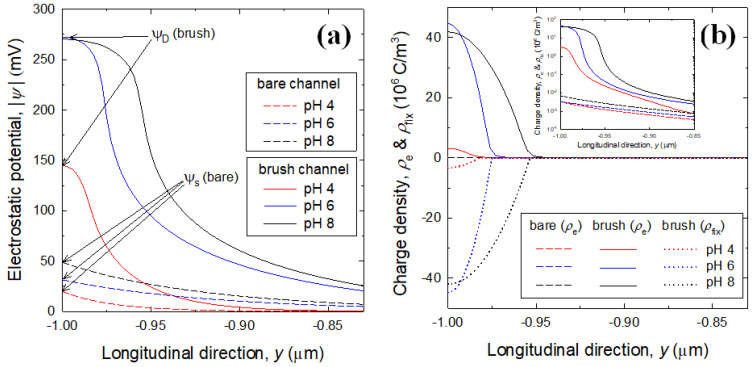
(**a**) Electrostatic potential profiles in the bare channel (dashed curves) and PAA brush channel (solid curves) filled with 0.01 mM KCl solution; (**b**) Corresponding charge density profiles, where the inset shows a plot on semi-logarithmic scale for visibility of *ρ_e_* of bare channel. Here, the PAA brush conditions are Mw = 30 kDa and *σ* = 0.1/nm^2^.

Electrostatic potential profiles and corresponding charge density profiles are presented in Figure 7a,b, where the electrostatic potential has a negative value since both PDMS and PAA are anionic. A simple fluid of KCl solution is filled inside bare and brush channels that account for rigid and soft channels, respectively. Here, the soft channel is represented by a rigid PDMS surface covered by a PE brush layer. For all pH conditions, the electrostatic potential in the brush channel is overall several times higher than that in the bare channel. Note that the potential profiles of brush channel show the form of Donnan potential (*ψ*_D_), similar to the situation of semipermeable porous membranes [41,42]. The formation of electrostatic potential by ion diffusion inside the brush layer is different from the surface potential (*ψ*_s_) at the impermeable surface in the case of bare channel, representing the Donnan potential due to the ion balance between inside and outside the brush layer. As same in the electrostatic potential, the charge density of brush channel is larger than that of bare channel, noting that its difference is extremely evident with several orders of magnitude. Although the charge density of bare channel is invisible in Figure 7b because its value locates very close to the *x*-axis, its difference increases with increasing solution pH. Note that, in the case of brush channel, the positive mobile charges in the bulk (*ρ_e_*) have approximately the same magnitude of the negative fixed charges (*ρ_fix_*) of PAA brush.

**Figure 8:**
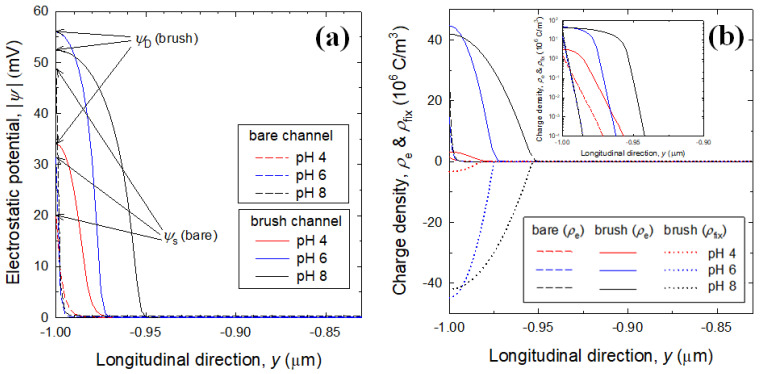
(**a**) Electrostatic potential profiles in the bare channel (dashed curves) and PAA brush channel (solid curves) filled with 0.4 wt% PAA solution; (**b**) Corresponding charge density profiles, where the inset shows a plot on semi-logarithmic scale for visibility of *ρ_e_* of bare channel. Here, the PAA brush conditions are the same as in Figure 7.

For bare and brush channels filled with 0.4 wt% PAA solution, electrostatic potential profiles and corresponding charge density profiles are presented in Figure 8a,b, respectively. In contrast to the channels filled with KCl solution, the ionization of PAA chain in the bulk solution leads to a certain increase in the total ion concentration, resulting in the reduction of electrostatic interaction in accordance with the thin EDL. Comparing Figure 7a and Figure 8a, the increased total ion concentration causes the Donnan potential to become lower. Contrary to electrostatic potential profiles, the brush channel exhibits mobile and fixed charge density profiles that are nearly consistent regardless of the bulk solution. This can be explained by recalling the fact that the amount of fixed charge is the same at the same pH and the equivalent amount of mobile counter-ions.

We expect surface and Donnan potentials to be verified potentially by experiments with either zeta potential or streaming potential measurements, and the force-distance curve by atomic force microscopy is possible to provide potential profiles. A complicated behavior of charge density should be identified in the interpretation of experimental data. This direction should be suggested as future studies. Quantitative predictions provide useful information that a variation in solution property gets nontrivial implications in the rational design and operation of microfluidic-chip with actual situations. As a final remark, our numerical framework can also be applied to analyze either pressure-driven or electric field-driven microfluidics, which is an ongoing study.

## 4. Conclusions

The PE brush-grafted microchannel, which is also filled with electrolytic fluids, has been analyzed by successfully developing a model framework based on the AdG equation for brush property and the PNP equations for ionic diffusion and migration. Considering ion transport in such a soft channel is significant for exploring either Newtonian or non-Newtonian microfluidics. Our explicit model fully takes into account the conditions of brush, bulk solution, degree of ionization, and each ion concentration. Our results of electrostatic potential profile estimated by the PNP continuum model was justified by comparison with the literature results determined by the PB equation.

Computation results reveal new information which was not reported before. The electrostatic potential in the PAA brush channel is everywhere several times higher than that in the bare channel, with accompanying Donnan potential. The charge density is also higher by gathering the counter-ions (i.e., cationic charges) in the bulk, according to anionic fixed charges due to ionization of the PAA brush. It should be emphasized that PAA bush is advantageous for possible enhancement of charge effect, but which appears to diminish in the presence of PAA solution. In contrast, the brush channel exhibits mobile and fixed charge densities that are nearly consistent regardless of the bulk solution. As aforementioned, an asset of this framework is to couple with flow fields, and PE brush-grafted soft channel is possible to have various applications, such as ion-transport based sensing, lab-on-chips, and current rectification.

## Data Availability

The data that support the findings of this study are available from the authors upon reasonable request.

## Nomenclature

As nomenclatures, the followings are used in this manuscript.

*b_K_*	length of Kuhn segment [m]
*C_i_*	molar concentration of ion species *i* [mol/m^3^]
*D_i_*	diffusivity of ion species *i* [m^2^/s]
*e*	elementary charge [C]
*F*_brush_	free energy of polymer brush [J]
*f*	degree of ionization of PAA chain dispersed in the bulk solution [-]
*f_b_*	degree of ionization of PAA brush [-]
*H*	channel height [m]
*h_b_*	brush height [m]
*I*	ionic strength [mM]
**J**_*i*_	flux vector of ion species *i* [mol/m^2^ s]
*K_i_*	mobility of ion species *i* [s/kg]
*k_B_T*	Boltzmann thermal energy [J]
*l_b_*	average distance between grafting points [m]
*N_A_*	Avogadro number [1/mol]
*N_K_*	number of Kuhn segments of a polymer chain [-]
*n_b_*	number of monomers in a PAA chain of brush [-]
*n_s_*	number of monomers in a PAA chain dispersed in the bulk solution [-]
Ka	acid ionization constant in the bulk solution [-]
Ka_*b*_	acid ionization constant in the brush [-]
***v***_*i*_	ionic velocity vector [m/s]
*x*, *y*	spanwise and longitudinal distances from channel centerline [m]
*W*	channel width [m]
Greek symbols	
*ε_o_*	dielectric permittivity of vacuum [C/V m]
*ε_r_*	relative dielectric permittivity of aqueous medium [-]
*κ*^–1^	electric double layer thickness [m]
*Λ_i_*	valence of ion species *i* [-]
*ρ_e_*	charge density in the solution [C/m^3^]
*ρ_fix_*	charge density in the brush layer [C/m^3^]
*ρ_e_^net^*	net charge density [C/m^3^]
*σ*	grafting density [1/m^2^]
Φ	monomer distribution function [-]
*φ*	normalized monomer distribution function [-]
*χ*	Flory–Huggins parameter [-]
*ψ*	electrostatic potential [V]
*ψ_D_*	Donnan potential [V]
*ψ_s_*	surface potential [V]
*ω*	excluded volume parameter [m^3^]
